# “I don’t like fitting a box, except the box I am”: genderqueer and nonbinary (GQNB) patient strategies for navigating healthcare

**DOI:** 10.1186/s12939-026-02906-y

**Published:** 2026-08-12

**Authors:** Shanaya Sidhu, Jennifer T. Anger, Victor Trasvina, Falisha Kanji, Kyle Okamuro, Sunny Mangiameli, Alan J. Card, Tara Cohen, Hanna J. Barton, the TRANS-SAFE Author Group

**Affiliations:** 1 School of Medicine, University of California, San Diego, USA; 2 Department of Urology, University of California San Diego Health, San Diego, USA; 3 Department of Surgery, Cedars-Sinai Medical Center, Los Angeles, USA; 4 Touro University California College of Osteopathic Medicine, Vallejo, USA; 5 School of Public Health, San Diego State University, San Diego, USA; 6 BerbeeWalsh Department of Emergency Medicine, University of Wisconsin-Madison, 800 University Bay Dr., Madison, WI 53705 USA; 7 Gender Health Program, University of California San Diego Health, San Diego, United States

**Keywords:** Transgender, Gender queer, Nonbinary, Health equity, Strategies

## Abstract

**Background:**

While genderqueer and nonbinary (GQNB) people comprise a significant percentage of the transgender community, very little research has explored the unique experiences and needs of GQNB patients. A critical challenge faced by GQNB patients is the degree to which health systems operate inside of a gender binary—as evidenced by the design of health technologies, organizational policies, and culture. Understanding how GQNB patients navigate health systems as they currently exist may give us insight into how to improve the design of those systems. This study aimed to identify GQNB patient strategies for navigating care.

**Methods:**

As part of the TRANS-SAFE study aimed at identifying system contributors to psychosocial safety of transgender and nonbinary patients, we conducted semi-structured interviews with GQNB patients (*n* = 21) and clinicians who care for GQNB patients (*n* = 23). We performed a deductive content analysis of the transcripts, applying a Human Factors Engineering-based work system model—The Systems Engineering Initiative for Patient Safety 2.0—and then reviewed coded excerpts to identify patient strategies, defined as “any dynamic response to perceived work system barriers to, or facilitators of, timely and effective performance of effortful work activities in pursuit of the patient’s health goals.” Strategies were summarized and grouped by “type” and “purpose” through an iterative, team-based process and work system barriers for each strategy were identified.

**Results:**

We identified four types of strategies that GQNB patients employed. These strategies included (1) “satisficing” (i.e., accepting a solution, even if unideal, that sufficiently satisfies one’s minimum requirements) around care, (2) leveraging tools/technologies or GQNB community expertise, (3) adjusting their behavior, and (4) taking ownership of their care. GQNB patients used strategies for five different purposes: (A) accessing care, (B) interacting with the health system broadly, (C) interacting with clinicians, (D) navigating clinical settings, and (E) interacting with the EHR.

**Conclusions:**

GQNB patients use a variety of strategies to ensure they receive appropriate and affirming care. These strategies point to the goals and needs of GQNB patients, yet primarily represent an attempt to receive the bare minimum in person-centered care. In fact, these strategies characterize patient work that is harmfully burdensome for this population. Studying the strategies leveraged by GQNB patients gives us insight into how we might improve health care for all patients.

**Supplementary Information:**

The online version contains supplementary material available at 10.1186/s12939-026-02906-y.

## Introduction

### Background

While genderqueer and nonbinary (GQNB) people comprise an estimated 43% [1] of non-cisgender individuals who fall under the transgender and nonbinary (TGNB) umbrella, very little health services research has explored the unique experiences and needs of GQNB patients. Existing research has largely focused on the experiences of transgender men [2], transgender women [3], or has studied the combined experiences of all TGNB individuals [4]. This leaves a critical gap in understanding the distinct needs and barriers faced by GQNB individuals in interacting with healthcare systems, resulting in material consequences for GQNB well-being [5]. To ensure equitable care for GQNB individuals, we must better understand GQNB patients’ current healthcare experiences and how their care meets, or fails to meet, their needs.

In a world that emphasizes the gender binary (i.e., the belief that human beings are categorized solely as women or men), GQNB people are routinely invalidated [6]. For example, many parts of language, such as honorifics (e.g., ma’am, sir), operate on a gender binary, contributing to frequent misgendering, i.e., use of a name, title, or pronoun that is incongruent with someone’s gender identity, of people who use they/them pronouns [7]. In a qualitative study with deductive thematic analysis using the gender minority stress model, nonbinary people across the United States reported “experiencing discrimination, rejection, harassment/violence, misgendering, internalized stigma, anticipated minority stress (i.e., the specific stresses a minority group faces in everyday life), and concealment (i.e., concealing aspects of a stigmatized identity to avoid discrimination), but in unique ways” [8]. From the minimal literature that does exist about GQNB individuals’ healthcare experiences, it seems that the healthcare setting is no different from day-to-day life. GQNB individuals are more likely than their cisgender counterparts to report that their clinicians had inadequate information to treat them, invalidated them, and/or pathologized them [9]. This harm is not only attributable to interpersonal errors like misgendering or dead-naming patients, but is the result of structural aspects of care [10]. The infrastructure of health systems—from intake forms, electronic health record (EHR) fields, and bathroom options, to provider education and even clinic names—often reflects a binary conception of gender. Thus, for GQNB individuals, simply interacting with health systems can produce harm by erasing identity or triggering prior experiences of discrimination. Re-designing our health systems to better serve this population requires an in-depth understanding of how GQNB people actively navigate and work around these binary infrastructures to secure the care they need.

### A systems approach

This study applies a sociotechnical systems lens, grounded in Human Factors Engineering (HFE), to examine the experiences of GQNB individuals in healthcare. Human Factors is the scientific discipline concerned with understanding the interactions between humans and the systems in which they operate, with the dual aim of improving human well-being and system performance [11]. In healthcare, this perspective supports analysis of how people, tools and technologies, tasks, environments, and organizational structures interact to shape care processes and outcomes [12, 13]. The modern perspective on patient safety views patient harm as *emergent from* the sociotechnical context of care; a result of complex interactions between system elements, not simply the result of “human error” [14]. Some HFE researchers are beginning to expand this to identify the system-based sources of inequity to better inform interventions to improve care that go above and beyond training staff [15–17].

One methodology for identifying system contributors to inequitable outcomes is through the study of workarounds, or *strategies*, as they are indicative of misalignments between the sociotechnical system and people’s goals. Prior research with families caring for children with medical complexity, as well as caregivers of older adults with dementia, has demonstrated the utility of exploring patient- and caregiver-generated strategies [18–20]. Applying this perspective to GQNB patient experiences allows us to move beyond individual interactions and examine the structural features of healthcare that systematically marginalize nonbinary identities. In doing so, we aim to identify not only points of failure, but also the adaptive strategies GQNB individuals use to secure care, revealing targets for more inclusive and equitable system redesign.

### Study objective

In this study, we sought to identify GQNB patient strategies for accessing and navigating health care.

## Materials and methods

### Parent study

This study is part of a larger multi-site, mixed-methods project aimed at identifying systems contributors to psychosocial safety of transgender and nonbinary patients, called TRANS-SAFE [21]. As part of the TRANS-SAFE study, we conducted observations in a variety of healthcare settings, interviews with TGNB patients and the clinicians who care for them, and reviewed patient safety events and patient satisfaction data. This study focuses on a set of interviews with genderqueer and nonbinary patients and the clinicians who care for them.

### Conceptual model – Systems Engineering Initiative for Patient Safety (SEIPS)

**Fig. 1:**
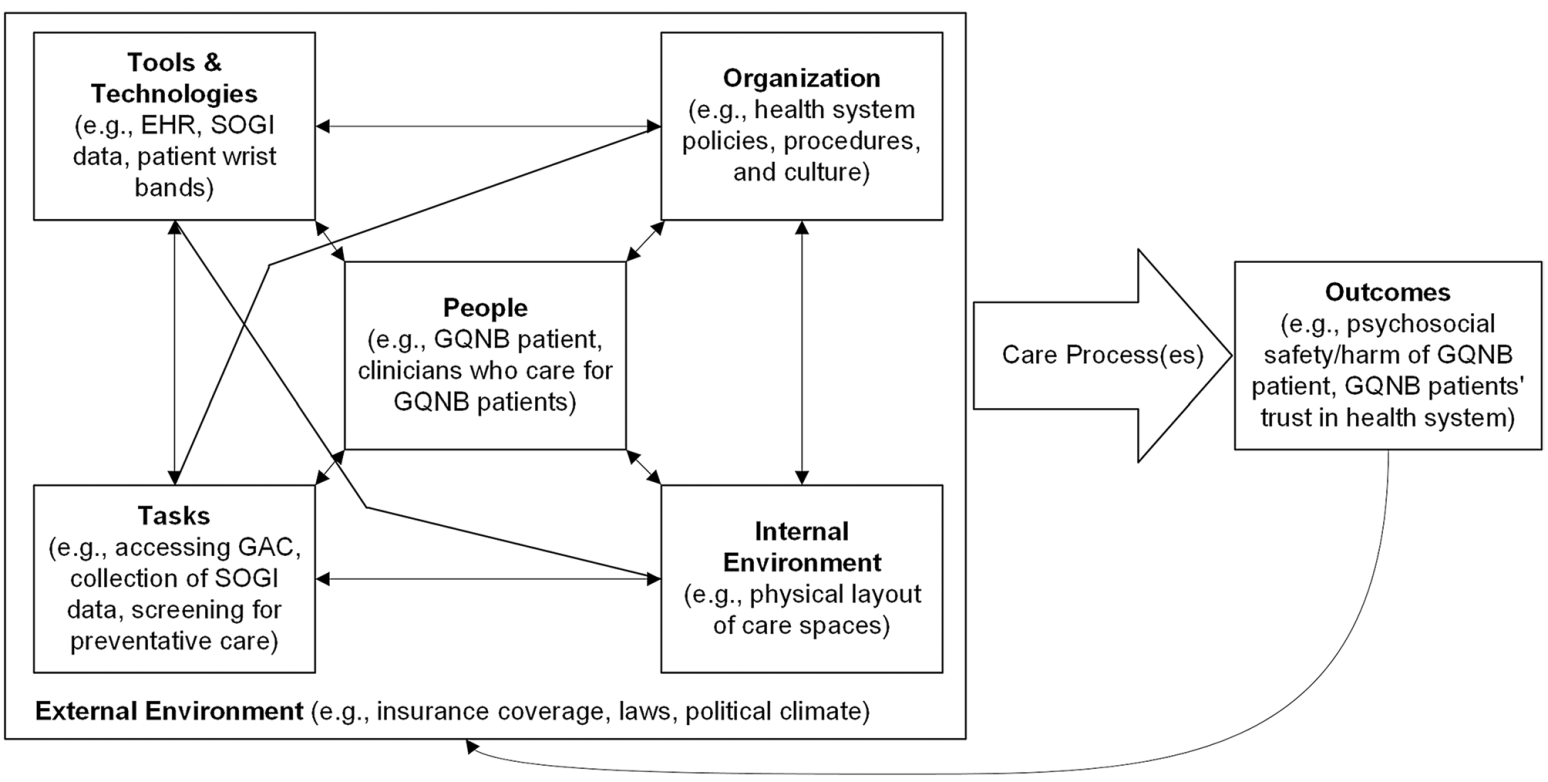
Systems engineering initiative for patient safety (SEIPS) 2.0 model with examples of work system components relevant to GQNB care. abbreviations: electronic health record (EHR), sexual orientation and gender identity (SOGI), gender affirming care (GAC)

For this study, we utilized the HFE-based Systems Engineering Initiative for Patient Safety (SEIPS) 2.0 model to distinguish between sociotechnical system components which included the *person(s)* involved in care, the *tasks* they conduct, and the *tools and technology* they use within their *organizational*, *internal*, and *external* environments (Fig. 1) [22]. SEIPS 2.0 posits that the resultant processes and outcomes are shaped by these sociotechnical factors and their interactions. It also provides a framework for thinking about how systems *adapt* over time, through feedback mechanisms from the outcomes and processes to both the processes and the work system. Namely, we conceptualize strategies as a type of *system adaptation*, “initiated by patients in response to perceived work system barriers to, or facilitators of, timely and effective performance of effortful work activities in pursuit of the patient’s health goals” [19].

### Recruitment

We recruited patients (n = 21) identifying as GQNB from various settings, including via the Gender Health Program of a large academic health system in the Western United States, social media outreach through local LGBTQ+ community organizations, and posting on GQNB social media groups. Inclusion criteria for patients included identifying as nonbinary, genderqueer or gender fluid and being at least 18 years old. We compensated recruited patients with a small $20 monetary gift card for participation. We recruited clinicians (n = 23) by emailing staff who were listed as gender affirming care clinicians on the institution’s, or affiliated clinics’, websites, as well as by searching for gender affirming clinicians on search directories, such as the OutList and LGBTQ+ Healthcare Directory. Clinicians were of various specialties and gender identities, including cisgender and nonbinary identities. This study was approved by the University of California, San Diego IRB as part of The TRANS-SAFE Patient Safety Learning Lab: Systems Improvement for Psychosocial Safety in Transgender Care [21].

### Data collection

We conducted semi-structured key informant interviews with patients and clinicians via video conferencing software (Zoom). Our interdisciplinary team, including members with expertise in Human Factors Engineering (HFE), sociotechnical and biopsychosociotechnical system analysis, human-centered design, gender-affirming care, qualitative research, and lived experience as GQNB individuals, created an open-ended topical guide that explored GQNB experiences, system barriers, coping strategies, and proposed improvements/solutions to these issues within healthcare (Appendix A). Interviews lasted an average of 45 min (min = 30 min, max = 90 min). Interviews were audio recorded and manually transcribed.

Prior to the interviews, we collected demographic information verbally to understand characteristics of the patient and clinician interviewees. Providing demographic information was voluntary. For patients, we recorded their self-reported age, gender identity, race/ethnicity, geographic location of residence, disability status, and insurance status. For clinicians, we recorded self-reported specialty, geographic location of practice, years of practice, gender identity, and race/ethnicity. Demographic data were compiled for basic descriptive statistics of the sample patient and clinician populations.

### Data analysis

*SEIPS Coding.* We conducted a deductive content analysis [23], applying the HFE-based work system model SEIPS 2.0 to both patient and clinician interview transcripts [22]. The codebook included the following codes: person (patient), person (clinician), task (patient), task (clinician), tools and technology, organization, internal environment, and external environment (Appendix B). Interview transcripts were uploaded to Dedoose (Manhattan Beach, CA, 2025 Sociocultural Research Consultants, LLC) and one researcher (SS) applied the codebook with training and consistent review by a senior research team member with extensive experience applying SEIPS (HB). Disagreements in coding were resolved through discussion. Transcripts were coded during the recruitment and data collection period, allowing for analytic saturation to be assessed when patient and clinician interviews were no longer yielding new themes or insights, as determined through iterative coding and constant comparison to assess whether additional interviews contributed novel codes or concepts. Coded excerpts were exported to Excel (Redmond, WA, Microsoft) for further analysis to identify strategies.

*Strategy Identification.* To identify strategies that GQNB patients employ, we applied the definition of a patient strategy outlined in Sect.  2.2. One researcher (SS) coded every exported excerpt from the patient and clinician interviews as a “strategy” or “not a strategy.” As a quality assurance step, another researcher (HB) reviewed every excerpt initially coded as “not a strategy.” From the initial review (SS), 61 patient strategies were identified. From this second review (HB), 17 additional strategies were identified, which account for 21% of the total patient strategy data set. Thus, in total from both reviews, 78 patient strategies were finalized. Of the patient strategies identified from the coded excerpts, three came from clinician interviews and 75 came from patient interviews. Given few patient strategies were reported by clinicians, clinician interviews informed contextual understanding but were not the primary analytic focus of this manuscript. The analysis team (SS, HB) then summarized and grouped the coded strategies by “type” and “purpose” through an iterative, team-based process [24]. Strategy types and purposes were then presented to and refined based on feedback from the large research team. Finally, for each of the 78 patient strategies, the work system barrier was identified and summarized by one researcher (SS) and reviewed and refined by another (HB).

## Results

### Demographics

Demographics of GQNB patients and clinicians are reported in Table 1. The average age of GQNB patients in this sample was 32 years. Patients’ self-reported gender identities included nonbinary, trans masculine, trans femme, gender fluid, and non-conforming. Patients were located in various states, including California, Wisconsin, Connecticut, Florida, Texas, New Hampshire, Illinois, Ohio, and New Jersey. Of the 21 patients, 76% reported living with a visible and/or non-visible disability and 90% of patients reported having insurance coverage.

Of the 23 clinicians in the total sample, three clinicians reported strategies utilized by their GQNB patients. For these three clinicians, their specialties included primary care, HIV care, and plastic surgery. Other specialties in the total clinician sample include critical care, pediatric plastic surgery, infectious diseases, ear, nose, and throat surgery, endocrinology, reproductive endocrinology, obstetrics and gynecology, psychiatry, speech language pathology, dermatology, and neurology. All of the clinicians in this dataset were based in Southern California, with an average of 12.7 years in practice. Two of the clinicians in the dataset identified as genderqueer, one of whom was nonbinary and the other nonbinary trans masculine.

### *GQNB strategies*

GQNB patients employed four types of strategies to navigate health care: **“satisficing”** (i.e., accepting a sub-optimal solution that sufficiently satisfies their minimum requirements) [25], **adjusting their behavior**, **taking ownership of their care**, and **leveraging tools/technologies or GQNB community expertise**. Adjusting behavior refers to behavioral modifications GQNB patients made to avoid negative aspects of care, while taking ownership of care refers to intentional choices GQNB patients made to create positive aspects of care. GQNB patients used these strategies for five purposes, which were to (1) access care, (2) interact with clinicians, (3) navigate clinical settings, (4) interact with the EHR, and (5) interact with the health system broadly. Table 2 lists the strategies employed for each of these purposes, on which we expand in the following sub-sections.

#### Strategies related to accessing care

GQNB patients detailed strategically navigating the entry points of healthcare and their associated barriers, deciding when and how to engage with their care. When GQNB patients faced issues with insurance coverage, they **satisficed** by *paying out of pocket for affirming clinicians* (*n* = 1) and *adjusting the length of their care* (*n* = 1). When one patient’s insurance coverage for their vocal coaching suddenly ended with no explanation, they pursued a payment plan and reduced their sessions in half to maintain a level of care they could afford.

On the other hand, another GQNB patient **took ownership** of financial aspects of their care by *finding more affordable avenues for affirming care* (*n* = 1), such as discounted vocal coaching provided by trainees.

GQNB patients used prior experiences of psychosocial harm in care settings, such as gendered clinic design (e.g., “women’s health” clinics), to inform when and how they access healthcare. For some patients, this meant **adjusting their behavior** by *avoiding healthcare* (*n* = 3). Alternatively, some patients **took ownership of their care** outside of traditional healthcare systems by *utilizing non-medical self-care strategies* to alleviate gender dysphoria, such as strength training or somatic work (*n* = 2).

For other GQNB patients, they aimed to minimize repeat occurrences of psychosocial harm by **taking ownership of their care** when choosing clinicians, such as by *seeking out clinicians with shared identities* (*n* = 2). Notably, Black and Brown GQNB patients experienced difficulties finding clinicians who shared both their gender identity and racial/ethnic identity. This led to patients having to prioritize one identity over the other.

Similarly, when choosing clinicians, GQNB patients **took ownership** by *vetting clinicians* (*n* = 7), i.e., doing extensive research to ensure they are affirming.

*I also have to struggle too with trying to read through everybody’s stuff and find doctors that I feel safe and comfortable with that are affirming…. I got really excited about one doctor that was listed on the page as offering gender affirming care….[only to find out that]… they don’t do that anymore. (P8)*

Geographic area of residence also played a role in how GQNB patients access care, necessitating they **take ownership of their care** by *traveling to access care* when they lacked nearby affirming clinicians (*n* = 2) and *accounting for the political landscape* when planning care (*n* = 3). Patients described needing to account for anti-trans legislation based on their area of residence when deciding how, where, and with which clinician to access their care.

While navigating these various barriers to accessing care, patients relied on **tools/technology and GQNB community expertise**. GQNB patients reported *leveraging or providing support from/for others in the GQNB community* (*n* = 3). For example, patients described utilizing their own care connections to direct their GQNB friends to affirming clinicians, writing questions for their GQNB friends to ask their clinicians during appointments, and sharing information on what options exist for care and what to expect from different types of GAC.

Similarly, GQNB patients *utilized online clinician directories* to find affirming clinicians (*n* = 2) and *online and written media* (*n* = 3), including Facebook support groups, written guides, articles, Reddit, and university health system and clinic websites, *to acquire information and guidance on GQNB care*.

*… I have my little trans masc guide [book] here that I keep with me… to have more things spelled out*,* and then also as the reminder of other people sharing my experience too. (P13)*

#### Strategies for interacting with clinicians

In their interactions with clinicians, GQNB patients **satisficed** around care by *expecting and tolerating harmful experiences*, such as misgendering (*n* = 5), and *being understanding when their clinician made mistakes* (*n* = 3). Patients reported being understanding about clinicians misgendering them to a certain point, such as the first few appointments, or depending on the clinician’s exposure to GQNB patients (e.g., if they were their clinician’s first GQNB patient they would be more tolerant). Simultaneously, they often needed to set expectations to mentally prepare for future negative experiences while hoping for positive ones. These specific strategies allowed them to be realistic in setting and achieving their overall care goals to a satisfactory level even if some specific aspects of that care were sub-optimal.

*I’m emotionally prepared to be misgendered all day…. even going through the drive through*,* or even… when I get a call back from a doctor’s office*,* and then somebody leaves me a voicemail and it says Miss X on it. (P4)*

The strategy type that GQNB patients employed most frequently during their interactions with clinicians was **taking ownership of their care**. For example, GQNB patients *asserted their expertise (e.g.*,* about body*,* about healthcare)* (*n* = 2) and *negotiated with clinicians around GQNB specific care* (*n* = 3). These two strategies allowed GQNB patients to partner with their clinicians to navigate guidelines of care and care practices that are often more oriented to binary GAC.

For example, GQNB patients described the need to negotiate hormone levels with their clinician, for example through “microdosing”, since current standard HRT dosing is often designed for trans men and women without much guidance on dosing for people who fall outside of the gender binary.

*And I went there [to the clinic]*,* and I [said] ‘hey do you think I should try going on T [testosterone]?’ And after talking with a doctor… it turns out*,* hormones… will do things that you might not want*,* especially if you’re not a binary trans person…. And so my doctor at X [said]*,* ‘well*,* if you just want your voice to get deeper*,* you go to voice training. You shouldn’t be on a hormone that could potentially cause side effects that you don’t really want.’ (P16)*

GQNB patients also **took ownership of their care** when *practicing and modifying their communication with clinicians*, typically making it simpler or more direct, to effectively articulate their needs (*n* = 2). This strategy required that patients understand how the health system operates to fill in the clinician’s knowledge gaps about the delivery of GAC.

For example, one patient described asking specifically for an endocrinology referral for HRT when their primary care clinician continued to state they do not know how to address their GAC needs.

*… there’s times where I feel we have to practice what we say and how we say it… just to get the prescriptions…. the only way we can get those things is if we use certain language…. to fit a box*,* which is also annoying*,* because I don’t like fitting a box*,* except the box I am. (P12)*

Another way in which GQNB patients **took ownership of their care** was by educating their clinicians, either by *correcting heteronormative assumptions* about their sexual orientation (*n* = 2), or, more generally, *educating clinicians on the experience of being GQNB* (*n* = 2). Notably, patients described this experience even with clinicians who had explicitly worked in gender-affirming care settings.

*… I think the providers specifically [say]… ‘oh*,* you’re taking estrogen so how*,* how’s your chest growing?’ but I’m also on another medication [Raloxifene] that blocks Estradiol from growing breast tissue. But she forgets that so every time I go in…. And so I still feel like there’s this level of educating the providers on I’m not a binary trans person…. Even when I’m personally seeing two of the more respected gender specialists in the area.”(P3)*

Long-term, GQNB patients detailed *building longitudinal relationships with clinicians* (*n* = 1) to minimize the need to consistently educate different clinicians on the same aspects regarding their identity and/or care, thus optimizing their care outcomes.

#### Strategies for navigating clinical settings

The most significant barrier to navigating clinical settings for GQNB patients was a lack of gender-neutral restrooms. Often, GQNB patients **satisficed** by *using gendered clinic restrooms* (*n* = 5), accepting a sub-optimal solution to maintain physical and psychosocial safety while navigating binary clinic design (i.e., only male and female restrooms available). In these cases, some patients preferred utilizing the bathroom that aligns with their sex assigned at birth (e.g., GQNB person assigned female at birth using the women’s bathroom because they do not feel safe in the men’s bathroom), while one patient utilized whichever bathroom was less full.

On the other hand, some GQNB patients **adjusted their behavior** by *avoiding using clinic restrooms* entirely, such as using their restroom at home beforehand, to avoid needing to choose between binary restroom options (*n* = 2). GQNB patients note that when gender neutral restrooms were present, they were often less accessible, for example, located multiple floors away from their clinical care.

These examples of navigating binary clinic design demonstrate how patients must often recognize and dynamically respond to the ways work systems fail to be GQNB-inclusive. Thus, several patients reported maintaining a certain level of “gender stealth,” as one patient words it, by **adjusting their gender expression** in clinical settings. For example, one patient described *not mentioning gender* unless they felt it was relevant to their care or if there was a clinician with whom they felt their identity would be understood (*n* = 1).

Other patients described *“masking*” to avoid misgendering and increase the likelihood they would be perceived the way they would like to be perceived (*n* = 2). Masking, broadly, refers to a process where a minoritized group performs the identity of the majority group to confer the benefits of being perceived as belonging to that group. In this case, GQNB patients described adjusting their gender expression (e.g., attire, chest binding) to be perceived as nonbinary [26].

*… I tend to mask myself*,* and so trying to just feel I can present in more of a way that I hope to be addressed. And that’s not necessarily the healthiest way*,* I don’t think*,* but I try to make sure… even though it may not be as comfortable*,* I should definitely bind to go to this appointment… so I will try to dress in the way that I’m hoping to be perceived. And so people cannot just automatically place me in a category. (P2)*

Some GQNB patients noted the psychosocial harm that often inherently accompanies being GQNB in an in-person clinical setting (e.g., being called by their deadname or misgendered in a busy waiting room). In response, patients **leveraged technology**, specifically by *utilizing telehealth* (*n* = 2).

Alternatively, GQNB patients **took ownership of their care** by *bringing their support systems to their appointments* (e.g., wife, spouse) to advocate for them and mitigate any invalidation they experienced navigating in-person clinical settings (*n* = 2).

#### Strategies for interacting with the EHR

GQNB patients **satisficed** by *tolerating incorrect descriptions of their gender identity* (*n* = 3) and *inaccurate diagnosis codes* (*n* = 1) within their electronic health records (EHR) to maintain insurance coverage and/or access to clinicians who delivered the care they needed.

*… I was diagnosed with transgenderism for insurance purposes…. I had already come to terms… [with telling] people I’m trans all the time [for sake of simplicity]. (P12)*

GQNB patients categorized the reasons for these inaccuracies as either issues with clinician understanding of GQNB identities and care, limited options for gender identity and/or diagnosis codes in EHR, clinician assumptions that patients have a binary gender identity, and/or insurance requirements (i.e. assigning a diagnosis of gender dysphoria) for coverage or accessing care.

Given the frequency of inaccuracies regarding patients’ gender identities in their EHR, many patients **adjusted their behavior** by *avoiding reading their charts* (*n* = 1). One patient described experiences of clinicians using their correct pronouns during appointments but then misgendering them in the notes written in their chart. Thus, they had to decide when the benefit of reading a note to obtain their health information outweighed the potential psychosocial harm of feeling invalidated and unseen by a clinician who they thought was affirming of their identity during the visit.

Some patients sought to **take ownership of their care** and proactively mitigate misgendering in their notes by *advocating for their correct pronouns to be included in their medical record* (*n* = 2).

#### Strategies for interacting with the health system broadly

On a macro level, GQNB patients **satisficed** by *accepting less-than-satisfactory experiences with broader health systems as neutral or even positive* (*n* = 3). Patients described that if an experience was not overtly harmful, they accepted that experience as satisfactory. For example, if the care was “75% positive,” then they considered that a positive and acceptable interaction with the health system.

However, when health systems failed in providing affirming healthcare, some GQNB patients **took ownership of their care**. One strategy by which they did this was by *engaging in systems-level advocacy*, sharing constructive feedback with health institutions to improve their delivery of GQNB care (*n* = 2).

*… I’ve called in [to the institution] hundreds of times*,* emailed hundreds of times*,* went on social media hundreds of times…. because [the institution] has this whole program dedicated to making it safer for trans and nonbinary folks. Yet they’re not hiring our community…. Ask our Black*,* trans*,* nonbinary*,* disabled folks*,* ask the people who you don’t see representation. Look at who you’re serving*,* who is not present… in the room*,* and those are the people you need to be talking to. (P7)*

Similarly, one GQNB patient streamlined their own care within their health institution by *moving to a pharmacy that better integrated with their primary care clinic* to ensure their hormone prescriptions were filled appropriately (*n* = 1).

*… I take estrogen and testosterone and… Raloxifene. They [the pharmacy] stopped filling the estrogen…. I guess they assumed that… I have a phallus…. So*,* I’m actually in the last three weeks in the middle of switching all of my medications over to the pharmacy at the X clinic. Because if they have a question they can ask my provider directly. (P3)*

### Identified systems barriers

From the GQNB patient strategies, we identified systems barriers across the whole sociotechnical system, including those associated with patient or clinician factors, patient or clinician tasks, organization, internal environment, tools and technologies, and external environment (Table 3). The most frequently reported barriers include lack of clinician exposure to GQNB people (a clinician factor), difficulty finding affirming clinicians (another clinician factor), clinicians interpreting patients through a gender binary lens (a clinician task), and lack of gender-neutral clinic restrooms (an internal environment factor).

Patients’ responses to these barriers varied—for example, patients described five different strategies for addressing the barrier of clinicians interpreting patients through a binary lens. These included: preparing for and tolerating harmful experiences (e.g. misgendering); negotiating with clinicians around care; educating clinicians; adjusting gender expression; and not mentioning gender until relevant. Strategy purposes were largely consistent within SEIPS components, for example, patient and clinician factors corresponded primarily with accessing care and interacting with clinicians while tools and technology most frequently corresponded to interacting with the EHR. The most prevalent strategy type used to address work system components overall was “taking ownership over care.” More specifically, this strategy type was used most frequently to address barriers related to clinician and external environment work systems factors. The next most prevalent strategy type was “satisficing,” which was relatively evenly distributed across SEIPS components.

## Discussion

This study applied a sociotechnical systems lens to examine how GQNB individuals navigate healthcare systems not designed with their identities in mind. In doing so, we identified the adaptive strategies GQNB patients employ to reduce harm, anticipate barriers, and advocate for themselves in structurally binary environments. These findings highlight that GQNB patients are not passive recipients of care, but active system navigators working around systemic misalignments to secure necessary care.

### Structural sources of psychosocial harm

Our findings affirm that psychosocial harm in healthcare is not incidental, but often the product of entrenched structural features—ranging from EHR fields and documentation systems to physical environments and institutional language—that reinforce binary gender norms [8, 27] These design elements not only constrain clinicians’ ability to deliver inclusive care but also compel GQNB patients to take on significant emotional and logistical labor. Such adaptations point to a persistent misalignment between healthcare work systems and the identities and needs of GQNB patients [22].

Psychosocial safety, defined here as freedom from identity-based harm, warrants explicit integration into system design [28, 29]. Just as physical safety is engineered through alarms and infection protocols, psychosocial safety must be intentionally designed into workflows and infrastructure. The responsibility for maintaining safety should not rest solely with GQNB patients. Systems should be designed to minimize the likelihood of psychosocial harm occurring in the first place.

### Varying goals

The goals that drive these patient-led strategies are not monolithic. For some GQNB individuals, the objective is psychosocial survival: avoiding invalidation, misgendering, or traumatization. For others, the aim is to access affirming care—care that acknowledges and supports their identities, offers relevant medical guidance, and fosters long-term trust. Crucially, what participants described as affirming care diverges from conventional models developed for binary trans patients. While traditional gender-affirming care may emphasize access to hormones or surgery, GQNB patients prioritized relational respect, linguistic and clinical flexibility to support personalized care goals, and being recognized as whole people beyond binary categories.

This distinction is essential. Without understanding how GQNB people define affirming care on their own terms, redesign efforts risk reproducing the very marginalization they intend to address. A system designed for binary transition pathways cannot be assumed to meet the needs of those who live outside the binary.

### System redesign

Adopting a systems perspective repositions the source of harm from individual bias to structural design. Interpersonal harms such as misgendering are situated within—and often facilitated by—systems that preclude the accurate representation of nonbinary identities. Therefore, structural interventions are required, not just clinician-level education. These may include healthcare institutions providing ongoing clinician education [30], increasing clinician exposure to GQNB individuals [31], and deconstructing gendered notions in clinical frameworks [32]. Table 4 includes example interventions to address the most frequently reported work system barriers.

Interestingly, three of the most reported barriers were all associated with a clinician or their work (task). This may be because the broader systems barriers that shape GQNB patients’ interactions with clinicians are harder for patients to identify and articulate. Thus, these barriers are felt in the micro-level interactions they have with clinicians. For example, finding an affirming clinician may be hard due to lack of GQNB care topics in their clinical training curriculum, lack of inclusion of GQNB people in the healthcare workforce, and/or clinical medical frameworks often being designed and taught within a gender binary. This suggests that an important area of study may be the strategies that clinicians use to provide care for GQNB patients within systems that fail to support them.

The strategies used by GQNB patients do more than critique existing structures—they offer direction for redesign. Understanding how patients navigate, resist, and adapt reveals opportunities to align systems with real-world needs. For example, repeated points of identity erasure (e.g., deadname in patient charts, necessitating a trans masculine patient complete a pregnancy test) in intake workflows signal where more flexible and affirming data structures are needed [29]. Centering lived experience as a source of design knowledge can drive inclusive innovation—benefiting not only GQNB patients but all patients by increasing flexibility, autonomy, and respect.

### Limitations

Several limitations must be considered in interpreting these findings. First, while this study aimed to capture a range of GQNB experiences, many participants (52%) were recruited from the same geographic region (i.e., Southern California), which may reflect a relatively narrow set of healthcare systems, cultural dynamics, or institutional policies. As such, the strategies identified here may not fully represent GQNB experiences in other regions with different healthcare infrastructures or sociopolitical climates. Patients and clinicians in this sample, both those from Southern California and those not, note that experiences in California are distinctly different from other states with less political and social protection for GQNB people. Further, given the majority of GQNB participants were insured, the strategies reported offer limited insight into how GQNB people who are uninsured navigate systems of care and financial barriers. Finally, our sample included a significant number of participants who identified as disabled which, while offering a unique perspective into how disability intersects with gender identity in care settings, may bias our data towards more savvy patients and limit generalizability to the broader GQNB community.

Second, while participants described a wide variety of strategies, this study was not designed to assess the prevalence of those strategies across the broader GQNB population. Future research could explore the prevalence of specific strategies across intersecting identities (e.g., race, disability, immigration status), or how patients develop and evolve their use of strategies over time as they cycle through different types of care.

Additionally, despite recruiting clinicians as part of the broader parent study and including their interview data in this analysis, this study focused solely on the strategies that GQNB patients utilize to navigate care. Thus, very little data from clinicians is reported here—only the descriptions of patients’ strategies. Future work should explore whether the barriers identified by patients are the same, similar, or different from those that the clinicians experience in providing care for GQNB folks, and further, the extent to which clinicians are aware of the strategies that patients are employing to access and navigate care.

Methodologically, one limitation is that strategy identification relied primarily on a single primary coder. While the analytic process included secondary review of SEIPS and strategy coded excerpts by a separate researcher, it is possible that we missed excerpts and/or strategies that might have been identified had there been a second primary coder. Lastly, while the Human Factors lens provides valuable insights into system design, this approach may not fully capture the affective and embodied dimensions of gendered experience in clinical spaces. Further integration of critical gender studies and participatory methods could strengthen future design efforts.

## Conclusion

Understanding the variety of strategies GQNB patients leverage to access care, navigate clinical settings, and interact with clinicians, EHR, and broader health systems offers insight into the structural and systemic factors that create inequities in GQNB care. Continuing to build upon research that distinguishes the unique perspectives of people who exist beyond the gender binary can help deconstruct the ways in which healthcare development, delivery, and practice is gendered. Importantly, identifying these ways in which healthcare is gendered, and the strategies GQNB patients use to navigate this, reveals potential interventions to deconstruct and rebuild these aspects of healthcare. As a result, healthcare can be re-imagined and re-designed to better respect patient autonomy and identity outside of normative boundaries, ultimately improving care for all patients.

**Table 1:** Demographics of GQNB patients and clinicians who care for GQNB patients

	Patients (*N* = 21)	Clinicians (*N* = 23)
Age	32 (23–46)	*N*/A
Gender Identity		
- Nonbinary	12 (57%)	1 (4%)
- Non conforming	2 (9.5%)	0 (0%)
- Gender fluid	2 (9.5%)	0 (0%)
- Genderqueer	0 (0%)	2 (9%)
- Nonbinary Trans	4 (19%)	0 (0%)
- Nonbinary Trans Femme	1 (5%)	0 (0%)
- Nonbinary Trans Masc	2 (9.5%)	1 (4%)
- Cis woman	0 (0%)	12 (52%)
Cis man	0 (0%)	7 (30%)
Race/Ethnicity		
- Black	5 (24%)	0 (0%)
- White, non-Hispanic	6 (28.5%)	12 (52%)
- White, Hispanic	0 (0%)	3 (13%)
- Middle Eastern, North African	1 (5%)	1 (4%)
- Native American	2 (9.5%)	1 (4%)
- Latine	2 (9.5%)	0 (0%)
- Asian American	2 (9.5%)	4 (17%)
- Multiethnic	3 (14%)	2 (9%)
Living with a disability	16 (76%)	N/A
Insurance status		
- Private, employer-based	15 (71%)	N/A
- Public (Medical, Tricare)	4 (19%)	N/A
- Uninsured	2 (10%)	N/A
Clinical specialty		
- Primary care, Family Medicine, Internal Medicine	N/A	8 (34%)
- Critical care	N/A	1 (4%)
- Plastic surgery	N/A	2 (9%)
- Pediatric plastic surgery	N/A	1 (4%)
- HIV medicine	N/A	2 (9%)
- Infectious Diseases	N/A	3 (13%)
- ENT	N/A	1 (4%)
- Endocrinology	N/A	1 (4%)
- Reproductive Endocrinology	N/A	1 (4%)
- OB/GYN	N/A	1 (4%)
- Psychiatry	N/A	1 (4%)
- Speech language pathology	N/A	1 (4%)
- Dermatology	N/A	1 (4%)
- Neurology	N/A	1 (4%)
Years in practice		
- 0–5 yrs	N/A	3 (13%)
- 5–10 yrs	N/A	6 (26%)
- 10–20 yrs	N/A	11 (48%)
- 20 + yrs	N/A	3 (13%)

**Table 2:** List of strategies

Purpose	Strategy Type	Strategy	*n*	Example quotes
1. Accessing care	Satisficing	Adjusting length of care due to financial and insurance barriers	1	*“I can’t afford $200 a session for six weeks [for vocal coaching]*,* and so we had to cut that in half*,* and change the way that it was done…. I was able to go on a payment plan…. But we never quite got a reason why it [insurance coverage] was stopped.” (P16)*
1. Accessing care	Satisficing	Paying out of pocket because patient cannot find an adequate clinician covered by their insurance	1	*“… mental health providers who I think would be most in line with what I’m looking for don’t take insurance*,* and so that’s… been a barrier for me. I went through several therapists where I live*,* and I finally just have to resolve to… paying out of pocket and hoping that my insurance will make an exception…” (P20)*
1. Accessing care	Adjusting behavior	Avoiding healthcare to minimize harm and discomfort	3	*“And sometimes I do kind of want to put off going to appointments*,* or try to get the appointments just done virtually where I don’t have to go in office*,* so I don’t have to deal with all of the [gendered] wall art and infographics they have up.” (P11)*
1. Accessing care	Taking ownership of care	Choosing clinicians with shared identities	2	*“Or also let’s say you want to have someone that’s [nonbinary]*,* but they’re not of color*,* so it’s not always the full thing that you really want but overall*,* it’s okay…. I don’t really feel like I found the perfect one*,* but it’s still trial and error with therapy.” (P17)*
1. Accessing care	Taking ownership of care	Vetting clinicians to make sure they are affirming	7	*“I remember one of the times when I did a consultation and I did two different consultations*,* and one was with a well known gender affirming surgeon*,* and another one was just kind of one that… I did a loose vetting of…” (P6)*
1. Accessing care	Taking ownership of care	Finding more affordable avenues for affirming care	1	*“… I am currently applying to do vocal coaching as part of a couple people’s doctoral thesis or graduate capstones so that I can access that without having to go through my health plan.” (P3)*
1. Accessing care	Taking ownership of care	Traveling to access affirming care	2	*“But I’ve had*,* again*,* people say*,* ‘oh you understand*,* I don’t have to explain myself*,* you believe me.’ I have people who come from Long Beach and Goleta and really far locations to come see me for a 30 minute in person appointment. And they travel hours one way to come see me. And they do that because they want someone who understands them*,* someone who is going to affirm them and support them and know where they’re coming from when some of these things come up.” (C8)*
1. Accessing care	Taking ownership of care	Accounting for political landscape when planning care	3	*“I’m from the Midwest… I came into California expecting to have fully did my research and prepared with my list of questions*,* and mentally prepared for a lot of pushback. And I didn’t have that experience here.” (P8)*
1. Accessing care	Taking ownership of care	Utilizing non-medical gender affirming strategies	2	*“I train a lot of upper body and back to change the more feminine aspects of my appearance.” (P1)*
1. Accessing care	Leveraging tools/technologies or GQNB community expertise	Utilizing directories to find affirming clinicians	2	*“I know that there’s websites now*,* luckily that direct you*,* and you can kind of choose if you want a queer or nonbinary [clinician] or [clinician who] specializes in that. But a lot of the time it’s not actually people that identify with those identities. It’s just that they specialize in that.” (P17)*
1. Accessing care	Leveraging tools/technologies or GQNB community expertise	Leveraging (or providing) support from/for others in the community	3	*“For the longest time I wanted to become a patient advocate which I have basically been in the community just [asking] what do you need to know about your shit. So I write it down in a google doc like say this tomorrow in your clinic meeting… to get what you want.” (P10)*
1. Accessing care	Leveraging tools/technologies or GQNB community expertise	Utilizing social media or other online tools to access information on what to expect and how best to navigate GAC	3	*“And*,* literally that idea I didn’t get because I work in health care. I got that idea off Reddit*,* off*,* you know*,* Reddit slash trans health or something*,* right? And I make fun of my patients that go get all their information from Dr. Reddit. But I got the idea of getting my vocal coaching through it because I have no way of accessing it.” (P3)*
2. Interacting with clinicians	Satisficing	Expecting and tolerating harmful experiences (e.g. misgendering)	5	*“… but what I said to him was ‘I guess I sort of just hardened myself up and expect everyone to misgender me. And so I appreciate that you even said something*,* that you cared to get it right.’ And I think that is still probably true in the healthcare setting*,* which is really sad. It’s not the kind of care that I would want. Then*,* can you even call it care?” (P19)*
2. Interacting with clinicians	Satisficing	Being understanding about mistakes	3	*“… the first couple times*,* we ought to be forgiving*,* because nobody assumes the neutral. But after a while it’s just like*,* come on. This is session number 10. You should know this by now.” (P16)*
2. Interacting with clinicians	Taking ownership of care	Building longitudinal relationships with clinicians	1	*“… I had a longitudinal care relationship with my primary care there… [when I think about it]*,* was she affirming of my gender? I don’t know*,* but we knew each other*,* right? And there’s so many different aspects of who I am that she saw and worked with me about and on and that I don’t know that everybody either knows how to find a relationship like that and build that or understands the value of it… I think a lot of people my age interact with care much more transactionally versus relationally*,* and yet I found that relational aspect is so much more valuable.” (P19)*
2. Interacting with clinicians	Taking ownership of care	Correcting heteronormative assumptions made by clinicians	2	*“I might have to make it clear that… there’s no man in the picture.” (P9)*
2. Interacting with clinicians	Taking ownership of care	Educating clinicians on GQNB identities	2	*“… my genital surgery was very specific to nonbinary people. Right? So it’s sitting down and having those conversations… I feel like I have to take a further step in educating them because even though I am going to some of the best gender affirming providers in Southern California*,* their brains are wired to binary trans people.” (P3)*
2. Interacting with clinicians	Taking ownership of care	Negotiating with clinician around GQNB specific care	3	*“I feel super comfortable when my testosterone and my estrogen levels are both just below cis levels for both*,* which is kind of hard to get to and maintain*,* but man*,* it feels great. Right? And I’m able to do that easier since I don’t have any [endogenous] source of hormones anymore and I can just play with medication levels…” (P3)*
2. Interacting with clinicians	Taking ownership of care	Asserting one’s expertise (e.g.about body, about healthcare)	3	*“My profession definitely is the biggest impact*,* because I want to be seen and validated as a patient*,* and so I have definitely*,* in the past*,* gone and been*,* right off the bat*,* I’m a nurse*,* I want XYZ…. it’s my body. I know what’s going on in my body*,* and I have a health degree*,* and I know to some extent what could be going on.” (P13/C13)*
2. Interacting with clinicians	Taking ownership of care	Practicing and adjusting communication to explain needs to clinician	2	*“… there’s times where I felt like we have to practice what we say and how we say it… just to get the prescriptions…” (P12)*
3. Navigating clinical settings	Satisficing	Using gendered restrooms in clinic if no gender-neutral restroom is available	5	*“I still just use a women’s restroom there because I don’t have time to find this other [gender neutral] bathroom.” (P18)*
3. Navigating clinical settings	Adjusting behavior	Avoiding use of clinic restroom if no gender-neutral restroom is available	2	*“… when there are places like that [e.g.*,* clinics without a gender-neutral restroom]*,* I tend to feel less comfortable going to the bathroom*,* and so now I’m in a very uncomfortable position where I’m potentially holding myself from going to the bathroom just to avoid feeling uncomfortable.” (P2)*
3. Navigating clinical settings	Adjusting behavior	Adjusting gender expression to avoid misgendering	2	*“… I almost feel like I have to present a certain way*,* like I think about what I wear to my appointments*,* to try and be a little more intentional…” (P9)*
3. Navigating clinical settings	Adjusting behavior	Not mentioning gender unless relevant to visit to avoid negative treatment	1	*“When I go to clinics I don’t mention gender if it’s not relevant…. So then I would just navigate it as a trans man to get the least amount of questions…. I try to be stealthy [about gender identity] with the staff and administration until I meet the physician.” (P10)*
3. Navigating clinical settings	Taking ownership of their care	Bringing support system to appointment	2	*“I tend to go to appointments with my wife and so that not being an awkward moment [is an indicator how affirming a clinic is].” (P2)*
3. Navigating clinical settings	Leveraging tools/technologies or GQNB community expertise	Utilizing telehealth to avoid the in person care setting	2	*Moving to telehealth has been a huge weight off as regard to the stress of going into a doctor…. being able to just do it over the phone and just done with it. That’s… good for the stress levels*,* because… you feel like you have a little more control… it’s on your terms… (P15)*
4. Interacting with the EHR	Satisficing	Tolerating incorrect descriptions of gender identity in EHR to avoid additional challenges	3	*“And so the way my brain goes is*,* I want to make it easy for the people who don’t understand to understand. So*,* even though I don’t say nonbinary*,* I say fluid*,* I wouldn’t mind being labeled nonbinary for the means of simplicity.” (P5)*
4. Interacting with the EHR	Satisficing	Tolerating inaccurate ICD10 codes to access care	1	*“And she’s asking because she wants to put the right ICD code and I’m telling her I know the right ICD code just put hysterectomy as gender affirming care and it’s funded by the state you will find it. So she keeps saying to me ’I’ll just put sterilization I’ll just put sterilization I’ll just put sterilization.’ I’m like*,* okay just whatever you want*,* just whatever.” (P10)*
4. Interacting with the EHR	Adjusting behavior	Not reading notes to avoid seeing misgendering	1	*“The first time I read through my summary notes*,* it was hard for me to even read them*,* because all my pronouns were wrong*,* right? And so the process with me reading through my notes and seeing myself being misgendered over and over and over again*,* I couldn’t even read*,* I couldn’t finish reading the notes*,* because it was upsetting me.” (P4)*
4. Interacting with the EHR	Taking ownership of care	Advocating for correct pronouns in EHR	2	*“And so the next time I saw that psychiatrist*,* I [said]*,* ‘hey*,* can you put somewhere in my chart that these are my pronouns?’… And the psychiatrist [said]*,* ‘yeah*,* I’ll look into it*,*’ and never got back to me about it.” (P4)*
5. Interacting with health system broadly	Satisficing	Accepting less-than-satisfactory experiences as sufficient because they were not harmful	3	*“… you [the clinician] got a 90%*,* it’s still an A*,* it’s just the little stuff where [I think to myself] I get why that happened*,* though. Like*,* it’s just your upbringing. It’s ‘ma’am*,*’ ‘sir*,*’ and there really isn’t a neutral*,* I get it.” (P16)*
5. Interacting with health system broadly	Taking ownership of care	Sharing constructive feedback with healthcare system	2	*“And I’ve shared this with [institution] 1000s of times. I’ve called in hundreds of times*,* emailed hundreds of times*,* went on social media hundreds of times*,* and it’s not just [institution]. There’s a lot of other places.” (P7)*
5. Interacting with health system broadly	Taking ownership of care	Moving pharmacies to better integrate care with their clinic	1	*“They [the original pharmacy] stopped filling the estrogen. They only filled the testosterone…. they assumed that*,* you know*,* I have a phallus and they just*,* they refused to fill the estrogen. So*,* I’m actually… in the middle of switching all of my medications over to the pharmacy at the [institution-affiliated clinic].Because if they have question[s] they can ask my provider directly through the chat feature…” (P3)*
***Sum of Strategies***			78	

**Table 3:** List of Work System Barriers Driving Patient Strategies

Work System Component	Barrier (# of occurrences)	Patient Strategy (# of occurrences) [strategy purpose, strategy type]
*Person (patient)*	Limitations of the medical model in addressing gender identity and gender presentation discordance (2)	Utilizing non-medical gender affirming strategies (2) [accessing care, taking ownership of care]
	Patient and clinician power dynamics based on patient identity (e.g., race, gender identity, professional experience) (2)	Asserting one’s expertise (e.g., about body, about healthcare) (2) [interacting with clinicians, taking ownership of care]
	Intersectionality of gender identity and sexuality creating additional barriers to affirming care (2)	Correcting heteronormative assumptions made by clinicians (2) [interacting with clinicians, taking ownership of care]
*Person (clinician)*	Lack of clinician exposure to GQNB people (9)	Being understanding about mistakes (3) [interacting with clinicians, satisficing]
		Practicing and adjusting communication to explain needs to clinician (3) [interacting with clinicians, taking ownership of care]
		Educating clinicians on GQNB identities (2) [interacting with clinicians, taking ownership of care]
		Tolerating incorrect descriptions of gender identity in EHR (1) [interacting with the EHR, satisficing]
	Difficulty of finding clinicians with intersectional identities (2)	Choosing clinicians with shared identities (2) [accessing care, taking ownership of care]
	Difficulty finding affirming and understanding clinicians (16)	Building longitudinal relationships with clinicians (1) [interacting with clinicians, taking ownership of care]
		Accepting less-than-satisfactory experiences as sufficient because they were not harmful (3) [interacting with health system broadly, satisficing]
		Vetting clinicians to make sure they are affirming (7) [accessing care, taking ownership of care]
		Bringing support system to appointments (2) [navigating clinical settings, taking ownership of care]
		Utilizing directories to find affirming clinicians (2) [accessing care, leveraging tools/technologies or GQNB community expertise]
		Leveraging (or providing) support from/for others in the community (1) [accessing care, leveraging tools/technologies or GQNB community]
	Lack of GQNB clinicians (1)	Traveling to access affirming care (1) [accessing care, taking ownership of care]
*Task (patient)*	Difficulty knowing how to communicate nuanced needs to clinicians (1)	Leveraging (or providing) support from/for others in the community (1) [accessing care, leveraging tools/technologies or GQNB community]
*Task (clinician)*	Clinicians thinking about and interpreting patients through a gender binary lens (6)	Expecting, preparing for, and tolerating harmful experiences (e.g. misgendering) (1) [interacting with clinicians, satisficing]
		Negotiating with clinician around GQNB specific care (1) [interacting with clinicians, taking ownership of care]
		Educating clinicians on GQNB identities (1) [interacting with clinicians, taking ownership of care]
		Adjusting gender expression to receive better treatment (2) [navigating clinical settings, adjusting behavior]
		Not mentioning gender unless relevant to visit (1) [navigating clinical settings, adjusting behavior]
*Organization*	Lack of organizational infrastructure and guidance on how healthcare workers can support GQNB people (1)	Expecting, preparing for, and tolerating harmful experiences (e.g. misgendering) (1) [interacting with clinicians, satisficing]
	Lack of organizational infrastructures for incorporating GQNB experiences in healthcare design and delivery (2)	Sharing constructive feedback with healthcare system (2) [interacting with health system broadly, taking ownership of care]
*Internal Environment*	Gendered clinic design (1)	Avoiding healthcare to minimize harm and discomfort (1) [accessing care, adjusting behavior]
	Lack of patient privacy in clinic design (1)	Utilizing telehealth to avoid the in person care setting (1) [navigating clinical settings, leveraging tools/technologies or GQNB community expertise]
	Lack of inclusive and gender-neutral restroom options in clinics (7)	Avoiding or adjusting use of bathroom in clinic (7) [navigating clinical settings, adjusting behavior]
*Tools & Technology*	Difficulty finding online information on GAC, how to access it, and what to expect (3)	Utilizing social media or other online tools to access information on what to expect and how best to navigate GAC (3) [accessing care, leveraging tools/technologies or GQNB community expertise]
	Lack of clinician utilization of EMR aspects focused on patient gender identity (i.e., patient’s listed pronouns) (2)	Advocating for correct pronouns in EHR (2) [interacting with the EHR, taking ownership of care]
	Lack of inclusive / accurate ICD10 diagnosis codes (2)	Tolerating inaccurate ICD10 codes (2) [interacting with the EHR, satisficing]
	Misgendering in notes (1)	Not reading notes to protect self (1) [interacting with the EHR, adjusting behavior]
*External Environment*	Societal and cultural use of honorifics (1)	Expecting, preparing for, and tolerating harmful experiences (e.g. misgendering) (1) [interacting with clinicians, satisficing]
	GAC and medical frameworks designs based on a gender binary (4)	Negotiating with clinician around GQNB specific care (2) [interacting with clinicians, taking ownership of care]
		Practicing and adjusting communication to explain needs to clinician (1) [interacting with clinicians, taking ownership of care]
		Educating clinicians on GQNB identities (1) [interacting with clinicians, taking ownership of care]
	Lack of affordable affirming care (1)	Finding more affordable avenues for affirming care (1) [accessing care, taking ownership of care]
	Difficulty accessing care based on pre-op or GAC requirements (1)	Leveraging (or providing) support from/for others in the community (1) [accessing care, leveraging tools/technologies or GQNB community expertise]
	Variability in insurance coverage of GAC services (3)	Paying out of pocket because they can’t find someone adequate (1) [accessing care, satisficing]
		Traveling to access affirming care (1) [accessing care, taking ownership of care]
		Adjusting length of care due to financial and insurance barriers (1) [accessing care, satisficing]
		Moving pharmacies to better integrate care with patient’s clinic (1) [interacting with health system broadly, taking ownership of care]
	Influence of local and state political environment on availability and delivery of affirming care (3)	Accounting for political landscape when planning care (3) [accessing care, taking ownership of care]
	Challenges having GAC paid for due to gender marker discrepancies between insurance and healthcare institutions (1)	Tolerating incorrect descriptions of gender identity in EHR (1) [interacting with the EHR, satisficing]

**Table 4:** Potential Interventions to Most Frequently Reported Work System Barriers

SEIPS Component	Systems Barrier	Potential Interventions
*Person (clinician)*	Lack of clinician exposure to GQNB people	Increasing exposure to GQNB patients through standardized patient experiences in clinical training curriculum [31]
		Creating standardized didactics for clinical training programs on GQNB and trans care [29]
	Difficulty finding affirming and understanding clinicians	Creating standardized directories (such as through insurance) for finding clinicians who specialize in GQNB care
		Healthcare institutions utilizing markers / descriptors in clinician profiles to signify clinicians who are GQNB affirming
*Task (clinician)*	Clinicians thinking about and interpreting patients through a gender binary lens	Improving EHR design (e.g., visibility of patient pronouns and gender identity, utilization of organ inventory, standardized tabs / smartphrases to improve clinician history-taking and care of GQNB people)
		Institutional policies and education focused on GQNB patients
		Promoting training and recruitment of GQNB clinicians and healthcare workers
		Funding teams or departments in healthcare institutions focused on quality improvement of GQNB care
*Internal Environment*	Lack of inclusive and gender-neutral restroom options in clinics	Increasing amount and visibility of gender-neutral restrooms in hospital and clinic spaces
*Tools & Technology*	Difficulty finding online information on GAC, how to access it, and what to expect	Local, state, and federal health agencies developing standardized, evidence-based educational materials about GAC
*External Environment*	GAC and medical frameworks designs based on a gender binary	Reframing clinical practice and algorithms based on body parts versus specific genders (i.e., specifying cervical, uterine, breast, etc. health vs. broad umbrella of women’s health)
		Promoting clinical research that includes and centers GQNB people to better understand the health status and needs of GQNB people in order to provide individualized medicine
		Practicing open-minded, dynamic history taking (i.e., asking what sexual organs someone has and what types of sexual behaviors they engage in to provide more clinically useful sexual health counseling) [32]
	Variability in insurance coverage of GAC services	Standardizing the classification of GAC as medically necessary (versus cosmetic or elective) [33]
		Aligning coverage of GAC amongst different types of insurance (e.g., government-sponsored, private, employer-sponsored)
		Improving implementation of state and federal anti-discrimination policies in insurance coverage (e.g., regulating blanket exclusions, preventing denial of coverage for care typically associated with a specific gender e.g. pap smear)

## Supplementary Information

Below is the link to the electronic supplementary material.

Supplementary Material 1

## Data Availability

The datasets generated and/or analyzed during the current study are not publicly available due to participant privacy but are available from the corresponding author on reasonable request.

## Abbreviations

GQNB: Genderqueer and Nonbinary
TGNB: Transgender and Nonbinary
HFE: Human Factors Engineering
SEIPS: Systems Engineering Initiative for Patient Safety
EHR: Electronic Health Record
GAC: Gender Affirming Care
