# Supplementary material for: “I don’t like fitting a box, except the box I am”: genderqueer and nonbinary (GQNB) patient strategies for navigating healthcare
Source: Int J Equity Health. 2026 Aug 12;25:194. doi: 10.1186/s12939-026-02906-y (PMC13465386; doi:10.1186/s12939-026-02906-y)
Supplement: Supplementary file 1 [file 12939_2026_2906_MOESM1_ESM.docx]

**Appendix A: Interview Guide**

Topical Guide: GQNB Patients

We wish to improve the treatment of nonbinary individuals in the healthcare system. I wish to record what you say, but there will be no identifiers recorded, meaning that what you say will be kept completely anonymous.

1. Have you had any **negative** experiences while getting medical care that made you feel discriminated against or unsupported as a nonbinary person?
   a. How did this experience affect you?
   b. How did this experience impact your likelihood of returning for care?
2. Have you had any **affirming** experiences that made you feel well supported as a nonbinary person?
   a. How did this experience affect you?
   b. How did this experience impact your likelihood of returning for care?
3. What **barriers** have you run into in accessing care as a nonbinary person? (e.g., scheduling appointments, insurance, etc.)
4. What **environmental factors** might impact your experience in healthcare as a nonbinary person? (e.g., signs, privacy, clinic design, waiting room area, location of the clinic, thickness of walls).
5. Can you describe ways you think that health care for nonbinary individuals can be **improved**?

Topical Guide: Clinicians

1. Can you please tell me any **negative** health care experiences you may have witnessed for nonbinary individuals.
2. Can you please tell me any **positive** health care experiences you may have witnessed for nonbinary individuals.
3. Have you ever “misgendered” a patient? If so, how did it happen, how did the patient respond, and how did you handle it?
4. How does the design of the EHR serve as a barrier or facilitator to providing gender affirmation?
5. Can you describe ways you think that health care for nonbinary individuals can be **improved**?

**Appendix B: Codebook**

| *Person (patient)* | GQNB individual receiving healthcare, and their physical, cognitive, and social characteristics |
| --- | --- |
| *Person (clinician)* | Individual providing healthcare to non binary and genderqueer patients, and their physical, cognitive, and social characteristics |
| *Task (patient)* | Activities performed by patient while receiving healthcare, and the characteristics of those tasks |
| *Task (clinician)* | Activities performed by clinician while caring for GQNB patients, and the characteristics of those tasks |
| *Tools & Technology* | Electronic health records, technologies, tools utilized by patients and/or clinicians |
| *Organization* | Characteristics of how work is organized, structured, and completed within individual health systems |
| *Internal Environment* | Characteristics of the physical environment of the setting of healthcare, which impacts the quality of care and healthcare experience |
| *External Environment* | Macro-level societal, economic, and policy factors outside an organization |
| *Strategy* | Any dynamic response to perceived work system barriers to, or facilitators or, timely and effective performance of effortful work activities in pursuit of the patient’s health goals |
